# Telomere Dynamics in Congenital Heart Defects: Hidden Architects of Cardiogenesis?

**DOI:** 10.1155/humu/5547171

**Published:** 2026-10-26

**Authors:** Radha Joshi, Shaoni Nath, Sanjay Gupta

**Affiliations:** ^1^ Sri Sathya Sai Sanjeevani Research Centre Sri Sathya Sai Sanjeevani Research Foundation Navi Mumbai Maharashtra India; ^2^ Department of Life Sciences Sri Sathya Sai University for Human Excellence Kalaburagi Karnataka India; ^3^ Epigenetics and Chromatin Biology Advanced Centre for Treatment Research and Education in Cancer Tata Memorial Centre Navi Mumbai Maharashtra India tmc.gov.in

**Keywords:** congenital heart defects, gamete telomere length, one-carbon metabolism, telomerase, telomere

## Abstract

**Background:**

Congenital heart defects (CHDs) represent a significant global health burden, particularly in India, and are among the leading causes of childhood mortality. Despite growing insights into their multifactorial etiology, the molecular mechanisms linking maternal health to embryonic heart development remain elusive. Telomeres, the repetitive DNA‐protein structures that cap chromosome ends, are recognized as markers of cellular aging and also as regulators of early embryogenesis. This review examines emerging evidence connecting telomere biology with the risk of CHD in offspring.

**Scope of Review:**

This review is aimed at mapping the existing evidence on telomere dynamics in the context of cardiogenesis and CHD. It encompasses studies involving human subjects and experimental models, focusing on telomere length (TL), telomerase activity, and telomere dysfunction in cardiac development and CHD pathogenesis.

**Key Findings:**

We highlight the influence of environmental stressors on telomere dynamics and chromosomal stability in gametes and early embryos. Current evidence suggests that altered telomere dynamics may influence cardiomyocyte proliferation, cellular senescence, and genomic integrity, thereby contributing to aberrant cardiac development. Studies indicate potential associations between telomere shortening, impaired telomerase activity, and increased susceptibility to CHD; however, findings remain heterogeneous and fragmented.

**Implications:**

This review highlights the need to explore impaired parental telomere maintenance as a novel risk factor for improper chromosomal segregation and abnormal heart development. Understanding the role of telomere dynamics in cardiogenesis may provide novel insights into the molecular mechanisms underlying CHD. This could aid in identifying potential biomarkers for early risk assessment and open avenues for targeted therapeutic strategies aimed at modulating telomere function.

**Conclusion:**

Telomere biology represents a promising yet underexplored area in CHD research. This study is among the first to conceptualize impaired parental and embryonic telomere dynamics as a unifying mechanistic link in CHD pathogenesis. This review highlights the need for comprehensive and integrative studies to elucidate potential mechanistic links in order to translate these findings into clinical applications. Understanding this telomere–CHD axis may open a new frontier for periconceptional intervention and targeted maternal care to improve overall birth outcomes.

## 1. Introduction

Congenital heart diseases (CHDs) are among the common birth defects and leading causes of childhood mortality and morbidity, with a high prevalence of 9 per 1000 live births in India [1–3]. A most recent analysis depicts that CHD prevalence globally is 6.4 per 1000 live births as of 2021, with 4.18 million cases under five worldwide [1]. South Asia seems to have the highest number of CHD patients (1,039,362 CHD cases in children < 5 years), with India having the highest absolute number of CHD cases in children under five in 2021, indicating CHD as a national public health problem [1]. These structural abnormalities during embryonic cardiac development range from simple septal defects to complex life‐threatening lesions [2]. The cardiomyocyte specification and commitment happen in human gestation, between 16 and 19 days postconception. The beating of the tubular heart and circulation are established in 21 days. Morphological development of the heart is complete by the initial 8 weeks of conception [3]. Cardiac development is a well‐orchestrated process that includes complex signaling events and coordinated regulation of gene expression in differentiating cardiac cells [2]. Any errors at these intricate steps are susceptible to external disruptions, leading to CHD, making its etiology very complex with the interplay of several genetic and environmental factors [4].

Telomeres—the chromosomal end caps—and telomerase have been significantly researched upon in the context of cancers and aging‐related diseases. Human telomeres consist of repeated (TTAGGG)n DNA sequences are bound by the six‐protein shelterin complex, which promotes the formation of a protective t‐loop structure. This configuration protects the chromosome ends from DNA damage. In normal proliferating cells, telomeres gradually shorten due to incomplete replication of the lagging DNA strand; they shorten faster under oxidative stress, exonuclease activity, and other contributing factors [5, 6]. Because of the replication process, telomeres diminish in length with each cell division, serving as a “molecular clock” for the cell [7]. Telomere attrition triggers cellular senescence, marking the cell for eventual apoptosis, thereby giving a finite lifespan per cell. Notably, the shortest telomere within a cell is a critical indicator for the initiation of senescence. Despite this, most telomere measurement techniques assess average rather than shortest TL [5]. Telomere shortening, an important biomarker, is often utilized to signify cellular senescence in early embryogenesis [7]. Telomerase is a ribonucleoprotein with reverse transcriptase activity that hTERT encodes in humans and complexes with a functional RNA unit and other proteins. This enzyme elongates telomeres and prevents subsequent telomere shortening per cell division [8].

Cardiomyocyte proliferation is high in the early embryonic stage and declines as the heart develops. In the human heart, telomerase activity is recorded until the 12th week of embryonic development, coinciding with cardiomyocyte production from myoblasts by histological differentiation [9]. The findings suggest that any telomerase activity changes during this critical period could permanently influence myoblast fate, potentially resulting in abnormal cardiac morphogenesis. Certain environmental stressors, whether physical or psychological, have been associated with shorter telomeres [10]. Cellular senescence is characterized by a permanent cessation of the cell cycle and a proinflammatory secretory profile known as the senescence‐associated secretory phenotype (SASP), which can be triggered by DNA damage responses (DRRs) as a result of critically short TL [11]. Senescent cells are metabolically active with a complex biological role that is yet elusive. SASP consists of many secreted factors like cytokines, chemokines, remodelers, extracellular matrix proteins, and exosomes. This secretome triggers recruitment of immune cells to remove senescent cells. During embryonic development, tissues that are prone to birth defects show senescent cells, indicating the probability of ectopic senescence in embryonic tissues, potentially leading to birth defects [11].

The heart–placental axis results in the parallel development of the placenta and heart. This process involves many common molecules and genes, highlighting the synergetic relationship between these two organs [9]. Canonical Wnt/*β*‐Catenin signaling and folate metabolic pathway are crucial for placenta development, implantation, and heart development. Any errors in these pathways result in abnormal heart development [9]. There is a higher oxidative stress during pregnancy due to the higher metabolic demand [10]. Oxidative stress is one of the reasons inducing cellular senescence, and such senescence in the placenta and fetal membranes is important for term parturition [12]. But persistent and premature senescence and TL as a senescence inducer are implicated in multiple pregnancy complications [12]. Maternal leukocyte TL (LTL) and micronutrient status (e.g., ferritin) are significantly associated in pregnancy [13].

The role of telomeres in CHD pathogenesis is relatively not well explored, with a more obvious role of this biomarker in age‐related diseases. Maternal factors during the periconceptional period, including oxidative stress and inflammation, can affect TL, thereby increasing the likelihood of CHD in children [14]. Maternal influences such as obesity, smoking during pregnancy, diabetes, and exposure to organic solvents may increase the risk of CHD in offspring [15]. Recent research indicates that reduced maternal TL, a sign of biological aging, is connected to a higher likelihood of congenital cardiac outflow defects (CODs) in children [16]. Shortened telomeres in mothers may indicate accumulated oxidative stress and inflammation, potentially impacting embryonic growth and elevating the risk for CHD [17]. Investigating the connection between maternal TL and the risk of CHD could lead to preventive measures and interventions during the periconception phase [16].

Given the limited understanding of the role of telomeres and telomere‐associated proteins in abnormal embryonic development, this review is aimed at exploring the potential link between telomere‐induced cellular senescence during early embryogenesis and the onset of abnormal cardiogenesis. This review is anchored on the hypothesis that telomere dysfunction—mediated by genetic, epigenetic, and environmental influences—disrupts genomic stability and cellular proliferation during cardiogenesis, thereby contributing to CHD pathogenesis. By critically analyzing existing literature, it proposes a hypothesis connecting telomere dynamics to disrupted cardiac development. This synthesis of current evidence is intended to inform and guide much essential future research into the molecular underpinnings of CHD and the influence of telomere biology during early development.

## 2. “Uncapping” Telomeres

Telomeres are highly conserved tandem repeats specific to species, at the terminal parts of chromosomes, preventing loss of terminal DNA per cell division and maintaining chromosomal stability [18] [19]. Telomeric repeats in *Homo sapiens* comprise (TTAGGG)n (Figure 1). End replication problem per DNA replication cycle gradually shortens telomeres after each cell division [20]. Telomeric length in humans corresponds to 10–15 kbps [18]. The rate of TL shortening in humans is estimated as 24.8–27.7 base pairs per year. Faster telomere erosion increases the chances of DNA damage and eventual disease state [21]. In humans, TL is highly heritable, longer in women than men, and there is large interindividual variability in TL in individuals of the same age. A less‐than‐average TL in a cohort, compared with the normal majority, has been proven to be attributed to disease phenotypes in that cohort [20, 22]. Shelterin consists of six components, which include telomere repeat Factors 1 and 2 (TRF1 and TRF2), repressor/activator Protein 1 (RAP1), TRF1‐interacting nuclear Factor 2 (TIN2), adrenocortical dysplasia homolog (ACD, also known as TINT1/PTOP/PIP1 [TPP1]), and Protection of Telomeres 1 (POT1) [23]. The CST complex comprises three components: conserved telomere maintenance component 1 (CTC1), suppressor of CDC13 homolog (STN1), and TL regulation protein TEN1 homolog (TEN1) [24]. Amir et al. [23] described how mutations in these components reduce TL.

**Figure 1:**
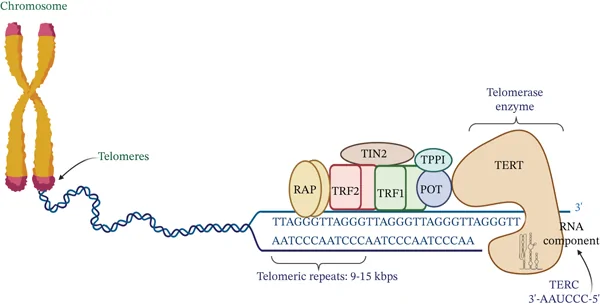
Structure of human telomeres.

### 2.1. Review Question and Search Methodology

Based on the premise that telomere dysfunction and associated proteins impair cellular proliferation, leading to genomic instability and aberrant cellular senescence during cardiogenesis and consequent pathological cardiac remodeling, this review is aimed at addressing the following question: How do telomere dynamics potentially influence cardiogenesis and contribute to the pathogenesis of CHD? A comprehensive literature search was conducted to identify studies examining the association between telomere dynamics and CHD. The search strategy included combinations of keywords related to telomere biology—such as “telomere length,” “leukocyte telomere length,” “telomere shortening,” and parental telomere attributes—with disease‐specific terms including “congenital heart disease,” “congenital heart defects,” and “outflow tract defects.” Electronic databases, including PubMed and PubMed Central (PMC), were systematically searched using Boolean operators, yielding a limited number of directly relevant studies. Additionally, Google Scholar was queried using an *all-in-title* search approach to identify articles with high relevance based on title‐specific matching. No restrictions were applied to the study design, and only articles published in English were considered. The search results were screened for relevance and gave us only two eligible studies.

### 2.2. Telomeres and CHD: Research So Far

The heart is almost a terminally differentiated organ with low repair and regeneration capacity [25]. It is the first organ to develop postfertilization, and heart development is a complex process tightly and intricately regulated at the molecular level. Any perturbations in this multilevel regulation may lead to abnormal cardiac development [2].

Fares et al. [14] carried out a multicentric case‐control study on a total of 730 mothers (306 case mothers of a child with a COD and 424 control mothers of a child without birth defects), distinguishing between those with CHD‐affected and unaffected offspring. This research analyzed maternal TL during the periconceptional window as a variable. Case diagnosis was based on ultrasound, cardiac catheterization report, or surgical findings. CHD associated with folate or any other environmental factors was included in cases to make a homogeneous group. Maternal blood samples were collected for analysis, around 15 months after the periconceptional period of the index pregnancy, to mimic the periconceptional period and avoid the risk of false negatives in controls in asymptomatic CHD lesions early postnatally [14]. This study also considered levels of biomarkers, such as homocysteine, serum folate, red blood cells, and serum vitamin B12 in participants.

The study reported comparable levels of homocysteine and folate between groups, whereas cases exhibited higher rates of preterm birth and lower birth weight. A mix of isolated and syndromic CHD phenotypes was observed. Importantly, shorter maternal TL was associated with an increased risk of ventricular septal defect (VSD) in offspring (OR = 1.29), which remained significant after adjustment for maternal age (adjusted OR = 1.25, *p* = 0.04). However, no significant association was identified between maternal TL and the overall risk of COD. This maiden study, though significant, faces certain limitations as even acknowledged by the authors. Stratification by CHD subtype reduces statistical strength and may contribute to inconsistent associations. Second, study design constraints, predominantly case‐control in nature, limit direct causal inference. Like many previous studies, this study relies on retrospective assessment of maternal TL, often measured months after the periconceptional period, which may not accurately reflect the true biological state during early embryogenesis. This introduces potential temporal misclassification bias. Third, there is a risk of selection bias and heterogeneity in case definitions, with inclusion of both syndromic and nonsyndromic CHDs, as well as variability in diagnostic criteria across centers. Additionally, confounding factors such as maternal age, nutritional status, environmental exposures, and socioeconomic determinants are not uniformly controlled across studies, potentially influencing observed associations. This study also has a notable underrepresentation of diverse populations, particularly from low‐ and middle‐income countries, limiting generalizability. Collectively, these findings suggest a CHD subtype–specific association between maternal telomere dynamics and CHD risk, rather than a uniform effect across all cardiac defects.

Current evidence remains limited, largely associative, and constrained by the lack of mechanistic insights, longitudinal data, and robust biomarker validation. The heterogeneity of CHD phenotypes further complicates interpretation, underscoring the need for integrative studies that combine genomic, environmental, and molecular data to elucidate potential causal pathways. Importantly, there is a critical need for focused investigations to unravel the underlying biological mechanisms linking telomere dysregulation to aberrant cardiac development.

### 2.3. Inheritance Pattern of Telomeres: Genetically Set or Epigenetically Tuned?

Although TL is consistent across various cell types within a single individual, it shows considerable variability between different individuals [26]. The researchers emphasized the inheritability of TL indicating that the genetic factors significantly influence an individual′s telomere behavior. A study indicated that the trait TL follows an X‐linked inheritance pattern [6], but subsequent research has proposed a paternal inheritance model for TL. A meta‐analysis that included more than 19,000 subjects found that the heritability of TL is approximately 70%, showing a stronger correlation between mothers and their offspring compared with fathers and their offspring, as well as a significant link between the age of fathers at conception and the TL of their children [27]. Similarly, a study focusing on the Old Order Amish community determined a heritability estimate of 44% for LTL, underscoring patterns of paternal inheritance and relationships with the parents′ lifespan [28]. On the contrary, a study examining Korean families over three generations revealed a heritability estimate of 0.64 for TL, with no notable patterns of inheritance related to gender [29].

Although some studies indicate a connection between paternal TL and that of their children, a strong estimate suggests that maternal inheritance plays a significant role in influencing the early life TL of offspring. A research study with 1031 Chinese participants from 108 families [30] discovered a strong correlation of TL between mothers and their children. In contrast, no similar association was observed between fathers and their offspring, indicating patterns of maternal inheritance [30], [31]. Studies suggest that the mother′s age at the time of conception may affect her offspring′s TL. A recent study identified a positive relationship between maternal age and the TL of offspring in the great reed warbler, a species of songbird [32].

A landmark study conducted a genome‐wide association analysis on 472,174 participants from the UK Biobank to investigate the genetic determinants of LTL. They identified 197 independent sentinel variants across 138 genomic loci significantly associated with LTL variation. Genetically shorter TL was causally linked to increased risks of several age‐related diseases, including cardiovascular disease, immune dysfunction, and pulmonary fibrosis. Notably, individuals with genetically determined shorter LTL (> one standard deviation below the mean) were estimated to have a 2.5‐year shorter life expectancy by age 40 compared with those with longer telomeres. Conversely, longer LTL was associated with elevated risk for several cancers, highlighting a biological trade‐off between longevity and tumor suppression. Many of the genes reported in this study have cardiovascular relevance (e.g., *RTEL1* and *POT1*). Mutations in *TERT*, *TERC*, *DKC1*, or *TINF2* genes are associated with Zinsser–Engman–Cole syndrome or dyskeratosis congenital (DKC), rarely coexisting with CHD [33]. These findings underscore telomere regulation′s polygenic and pleiotropic nature and provide a foundational resource for understanding how telomere biology influences human health and aging [34].

The genetic inheritability of telomeres is not the only factor determining TL dynamics in the zygote. A recent article gives a different perspective on telomeres as more of an epigenetic trait [35]. Gamete TL decides TL in the zygote. Yet TL is variable in gametes despite constitutive telomerase expression [36]. It is shown that grandparents (G0) with mutant telomerase may not display a discernible phenotype. However, shorter telomeres may not be maintained in the G1 generation due to multiple cell divisions, and thus, phenotypes may start to emerge in subsequent generations [35]. Animal studies found that in crossing heterozygous (*TERT* ^+/-^) parents, the offspring with *TERT* ^+/+^ had very short TL, though there was abundant telomerase. Thus, the findings indicate TL as an epigenetic trait influenced by additional factors, telomerase genetics, and the inheritance from our parents [37].

Another article reveals insights about shelterin protein dysfunction and embryonic developmental errors [38]. The shelterin complex prevents the DDR at telomeric ends by suppressing inappropriate repair of telomeres as double‐stranded breaks. Essentially, this means that aberration of the components of this complex would result in activation of DNA damage checkpoints (e.g., p53), leading to apoptosis or cell cycle arrest in progenitor cells. The role of shelterin proteins in maintaining telomere integrity implies critical involvement during early development, where cells are rapidly dividing, and telomeres must be tightly regulated to prevent chromosomal instability. Mutations in *TIN2 (TINF2)* and *ACD (TPP1)* are linked to dyskeratosis congenita (DC) and bone marrow failure (BM failure). These mutations cause stem cell exhaustion, impaired tissue renewal, and multisystem dysfunction, which are embryonically rooted processes and can plausibly lead to organogenesis defects, including heart, brain, and BM anomalies [38]. All these analyses suggest that TL is a polygenic and epigenetically regulated trait influenced by parental inheritance and genomic factors, with key roles in developmental homeostasis and disease susceptibility.

### 2.4. Telomere‐Associated Protein: Role of TERT in Embryonic Development

Telomere assortment in offspring is random, and telomeres differ significantly in length. TL is monitored and maintained by telomerase, and any aberrations in the *TERT* (gene coding for telomerase) sequence or expression lead to telomeropathies. Though not fully understood in terms of mechanism, the telomerase enzyme has distinct roles apart from telomere lengthening; as it is intricately involved in Wnt signaling, regulation of transcription, and hematopoiesis [37]. A report in 2009 uncovers a critical noncanonical role of TERT, the catalytic subunit of telomerase, in regulating Wnt/*β*‐catenin signaling independently of its telomere‐lengthening function. The authors demonstrate that TERT interacts with the chromatin remodeler BRG1 and *β*‐catenin, facilitating the transcription of Wnt target genes such as *Myc*, *Cyclin D1*, and *Axin2*. This interaction occurs at the promoters of Wnt‐responsive genes and enhances their transcriptional activity, contributing to stem cell function and early developmental processes. *TERT* overexpression mimics Wnt signaling effects in vivo, as seen in Xenopus embryos and murine models, and *TERT*‐null mice exhibit defective Wnt signaling responses with homeotic changes in vertebrae. This study provides strong evidence for a positive feedback loop where Wnt activation induces *TERT*, which augments Wnt signaling, although TL differs substantially between humans and rodents (here, mice). Future studies are required to determine the extent to which these differences may limit the direct translation of findings from murine models to human disease contexts, including CHD. Yet, this study significantly extends the understanding of the extratelomeric roles of telomerase, positioning TERT as a transcriptional coregulator in developmental and cancer‐related pathways [39].

During early embryonic development, the first step in cardiogenesis is defined by regulating mesodermal commitment to cardiac lineage by the Wnt pathway through *β*‐catenin‐dependent signaling [25, 40]. At later stages, canonical Wnt signaling inhibits cardiogenesis, highlighting its stage‐specific and biphasic role in cardiomyocyte differentiation. However, the noncanonical role of this pathway is also found in cardiogenesis [25]. Hyperactivation of this pathway has been shown to lead to heart defects in frogs. Also, loss of this signaling results in randomization of heart tube looping [25]. Even minor changes in the expression of Wnt‐responsive cardiac markers have been linked to various CHDs in both animal models and humans [40]. Together, these findings position telomerase, particularly TERT, as a pivotal regulator at the crossroads of telomere biology and Wnt‐mediated cardiac development, where its dysregulation may silently orchestrate the earliest molecular origins of CHD.

### 2.5. Telomere‐Linked Regulation Patterns During Embryogenesis

#### 2.5.1. Zygotic Genome Activation (ZGA) and Telomeres

The longer lifespan in women with extended fertility was established in epidemiological studies. Recent research reported that longer LTL indicates better oocyte quality and augmented chances of successful conception with healthy birth outcomes [41]. Levels of anti‐Müllerian hormone (AMH), which is a surrogate marker of oocyte reserve in women, have been directly correlated with shorter TL in mothers, especially with advanced age [41]. It was found that telomere‐specific mutations in generation F0 might not affect the same generations but lead to reduced TL in gametes. Maintaining this short TL during postfertilization embryonic development may be difficult, leading to disease phenotypes. This telomere shortening happens progressively in subsequent generations [35].

ZGA is a critical stage during early life when the embryo begins to transcribe its genome and stops relying on maternal mRNAs and proteins to set up totipotency. Initial low transcription levels (minor ZGA) are later taken over by a burst of transcription activity called major ZGA. *DUX4/Dux* expression is the initial key inducer of ZGA [42]. In humans, DUX4 is situated in the subtelomeric region of Chromosome 4 at 4q35.2, within a D4Z4 macrosatellite repeat array consisting of roughly 10–100 units, and encodes a homeodomain‐containing transcription factor [42, 43]. In adult patients with facioscapulohumeral muscular dystrophy (FSHD), *DUX4* expression in myotubes and myoblasts is inversely proportional to TL, possibly indicating release of telomere position effect (TPE) due to progressive age‐related telomere shortening. A study proposes that telomeres regulate DUX4/Dux through chromatin remodeling, contributing directly to ZGA regulation [42]. Short telomeres (seen in telomeropathies or maternal stress) may derepress *DUX4*, leading to ectopic expression. Persistent *DUX4* expression has been shown to repress differentiation and trigger apoptosis in embryoid bodies and stem cell models [42, 43]. Thus, critically short telomeres may reduce TPE, leading to earlier or inappropriate *DUX4* expression. Since DUX4 initiates ZGA, misregulation could perturb early embryonic transcriptional programs. It may disrupt maternal mRNA clearance, pluripotency establishment, and lineage specification. These findings may outline the hypothesis that aberrant ZGA may be linked to developmental arrest or congenital anomalies through disruption of the balance between cell division and apoptosis. Figure 2 illustrates that compromised gamete quality, driven by telomere dysfunction and genomic instability, can impair early embryonic development, leading to defective cell division and organogenesis. This cascade may ultimately contribute to developmental abnormalities, including CHD, through disrupted molecular and cellular pathways.

**Figure 2:**
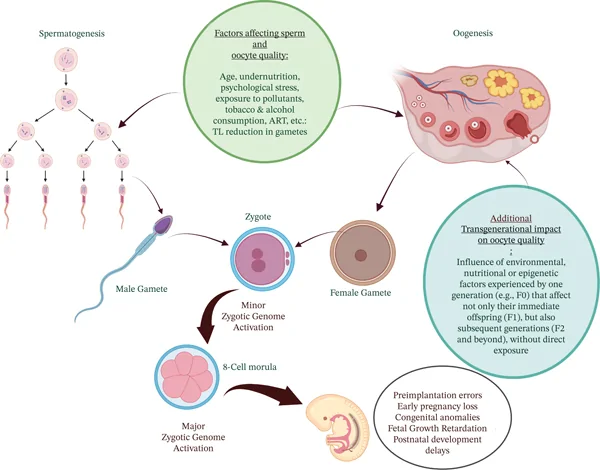
Theory of compromised gamete quality and downstream embryonic development.

#### 2.5.2. Gamete (TL) and Post‐fertilization Impact

Shorter LTL and mutations in telomere‐associated genes are associated with a series of telomeropathies and congenital defects, including dyskeratosis congenita, pulmonary fibrosis, aplastic anemia [44], and congenital limb abnormalities [45]. In humans, spermatogenesis continues for life, requiring a balance of mitotic and meiotic functions. Telomeres are essential for genomic stability, especially during meiosis [46]. Sperm TL (STL) is shown to prolong life, whereas LTL decreases with advancing age. A recent report investigated variation in newborn TL in the context of multiple periconceptional environmental factors. The study showed a positive association of newborn TL with parental age, with paternal age having a dominant effect. Babies of older mothers had longer telomeres. Also in such babies, paternal age was higher too. This can be correlated to an increase in STL with advanced age of fathers and intergenerational plasticity of TL [47]. This study emphasizes the effect of genetic variants at 3q26.2, maternal TL, paternal age, and maternal antenatal health on newborn TL [47].

In contrast, at around the 6th month of conception in females, there is minimal telomerase activity in oogonia and resultant arrest of oocytes in prophase of the first meiosis [46]. In women, reproductive aging is reflected in continued oocyte atresia, leading to a gradual decline in follicular reserve. This aging is more linked with diminished oocyte quality, resulting in higher rates of aneuploidy and miscarriages. Shorter LTL and higher epigenetic age based on leukocyte DNA methylation are shown to be associated with aneuploidy, premature ovarian insufficiency, and potential idiopathic infertility [48]. Though this indicates that LTL can be explored to predict the reproductive decline in ART, there are very limited data to emphatically state this association, requiring further in‐depth research [46].

A study showed that exposure to a low‐protein diet in the F0 generation depletes the ovarian reserves of the F2 generation with increased intra‐abdominal fat mass, higher oxidative stress, DNA copy instability, and faster erosion of telomeres in rats [49]. This study indicates that a suboptimal maternal diet may lead to reduced oocyte quality, contributing to generational effects. Human oocytes are very much prone to the erroneous segregation of chromosomes during meiosis and the resulting genomic instability, one of the leading causes of spontaneous loss of pregnancy or adverse phenotypes in newborns (e.g., miscarriages in fetuses with Trisomy 16) [50]. Using a parallel approach of telomere DNA quantification and 24‐chromosome aneuploidy screening from the same oocyte or embryo biopsy, the researchers found that aneuploid human polar bodies exhibited 3.07‐fold lower telomere DNA than their euploid counterparts (*p* = 0.016). Similarly, aneuploid embryonic cells had 2.60‐fold lower telomere DNA at the cleavage stage (*p* = 0.002). However, this difference was insignificant at the blastocyst stage (1.18‐fold, *p* = 0.340), suggesting normalization of TL or selection against severely affected embryos. This study shows that telomere shortening contributes to aneuploidy in oocytes and during the early implantation stage of the embryo, explaining the improper chromosomal segregation during division of oocytes [50]. Such errors during early embryogenesis, triggered by short TL, may lead to genomic instability that can disrupt tightly regulated cardiogenic pathways, thereby contributing to abnormal heart development.

## 3. Discussion

### 3.1. Assessing the Knowledge Gap

This review presents limited evidence indicating that maternal TL may be predictive of fetal cardiac development outcomes. This association suggests maternal genomic stability, cell signaling pathways, oxidative stress status, and telomere dynamics could influence or mirror fetal cardiac development. Figure 3 depicts how gamete telomere dysfunction induces genomic instability, altered cell proliferation, and premature senescence during organogenesis, disrupting key developmental pathways. These perturbations impair cardiac septation and morphogenesis, contributing to the development of CHD.

**Figure 3:**
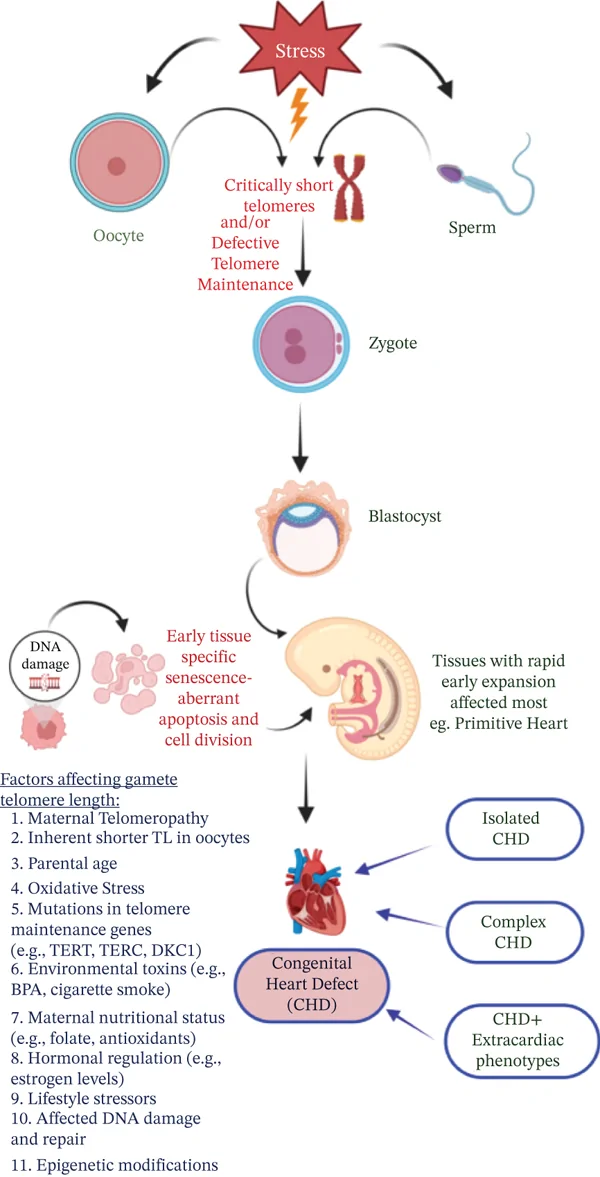
Proposed mechanism of telomere‐mediated abnormal cardiac development.

Longer TL in mature germ cells than somatic cells has been reported previously. However, TL regulation during embryogenesis is not completely known. TL establishment during embryogenesis will determine telomere reserves in newborns. By a telomere‐dependent mechanism, TL is decided at the morula to blastocyst transition [7]. The telomere theory of reproductive aging suggests that age‐related oocyte dysfunction in women is due to progressive TL shortening [51]. An experiment in mice showed that TL, indicative of human reproductive aging, resulted in asymmetric spindles and abnormal chromosome congression. Such embryos further resulted in chromosomal abnormalities; they stopped dividing and became fragmented [51]. Previous studies present an adverse antenatal history in terms of frequent miscarriages in mothers with shorter TL as compared with age‐matched healthy controls [51, 52]. Age‐related maternal loss of fertility is attributed to critically short telomeres in oocytes, which may be a result of late exit from oogonial cell cycles during fetal life, leading to shorter telomeres in adulthood and also exposure to reactive oxygen species (ROS) from prolonged arrest in the adult ovary [52]. Paternal telomeres contribute to longer telomeres in embryonic cells. Paternal telomeres elongate with age. Based on several previous studies, the theory of paternal telomere inheritance indicates that the older the father′s age, the higher the TL in offspring [32]. Maternal inheritance of TL has also been equally explored and resulted in the association of maternal factors, including age, with TL in offspring [32]. To summarize, several maternal and paternal genetic and other factors and environmental exposures determine the TL in offspring [32].

TL studies in newborns showed significant synchrony among WBCs, umbilical artery cells, and foreskin. This study also showed no difference in TL measured by TRF in male and female newborns [22]. This study showed that TL in skin cells was much less than in WBCs. Though the biological explanation of this observation is not straightforward, it may be speculated that skin comprises a highly proliferative base layer, which may result in near‐senescent cells in the epidermis. In contrast, WBC turnover varies from hours to months depending upon several cell subpopulations and stressors like inflammation. Compared with these cell subpopulations, cardiac tissue is relatively stable postearly embryonic life; cardiomyocytes are largely postmitotic and are expected not to show fast TL attrition unless disease phenotype sets in. Yet, TL has been explored as a biomarker in coronary heart disease to predict progressive damage to cardiac tissue [53].

The genetic and epigenetic regulation of telomeres remains a highly compelling area of investigation. Prior studies, such as Broer et al. [27], have demonstrated greater TL concordance among monozygotic twins than dizygotic twins, underscoring a strong genetic influence on telomere biology. These findings support the need to examine variants in *TERT* and other telomere‐associated genes for their potential roles in shaping early‐life TL and influencing embryonic developmental trajectories. Concurrently, various forms of intrauterine stress—including maternal, placental, and fetal immune, endocrine and oxidative challenges—have been implicated as potential mechanisms contributing to telomere programming during early development [54]. Certain animal studies have shown links between maternal nutrition alterations and tissue or organ‐specific changes in embryonic TL. Increased risk of preterm birth is linked with lower maternal TL [54]. Maternal telomere shortening, potentially due to oxidative stress, inflammation, or environmental factors, could hinder fetal heart development with a mechanism similar to the above‐illustrated maternal reproductive aging and adverse pregnancy outcomes. Thus, maternal health is critical for fetal telomere inheritance and subsequent cardiovascular risk [20]. Similar observations have been confirmed in a study that gave evidence for the interaction of genetic and environmental factors determining the association of maternal TL with her metabolic health and nutrient status, leading to a transgenerational effect on TL of newborns [47].

After zygotic gene activation during preimplantation embryonic development, kilobases of telomere repeats get added even in telomerase null mice by alternative lengthening of telomeres (ALT) [52]. However, telomerase‐dependent telomere dynamics in mouse experiments suggested that telomerase gene‐deficient embryos are postnatally presented with neural tube defects [55], suggesting that ALT‐mediated compensation may be incomplete. Collectively, these findings indicate that early embryonic development is highly sensitive to telomere dysfunction, which can lead to genomic instability and developmental defects [55].

Folate deficiency and heightened oxidative stress and inflammatory responses contribute significantly to telomere shortening. It has been shown that combinations of B‐complex vitamins and folic acid improve IVF outcomes [56]. Folate is a naturally occurring antioxidant and is an important substrate of OCM [14]. The pathway underpins essential cellular functions such as proliferation, differentiation, redox defense, amino acid homeostasis, DNA methylation, biosynthesis of purines and pyrimidines, and DNA repair; all of which rely heavily on folate, vitamin B12, and homocysteine as key metabolites [57] [58] [59]. Folate (B9) belongs to the water‐soluble vitamin B‐complex and is an important cofactor in OCM. The vegetables and pulses in the diet or synthetic supplements contribute to folate [60], which is essential in the first trimester when organogenesis occurs. Since folate demand increases during pregnancy, folate deficiency is established to be closely associated with fetal congenital anomalies [61].

The ratio of *S*‐adenosylmethionine (SAM) to *S*‐adenosylhomocysteine (SAH) serves as an indicator of a cell′s methylation potential, with elevated SAH exerting inhibitory effects on methyltransferase enzymes and thereby impeding gene regulation, programmed cell death, and differentiation [62] [63]. Homocysteine accumulation leads to a corresponding rise in SAH levels, strongly suppressing methylation processes. Folate and homocysteine levels are influenced by dietary intake and genetic factors, including polymorphisms [64, 65]. Elevated homocysteine is linked to DNA hypomethylation [66], a condition implicated in the pathogenesis of congenital heart defects like tetralogy of Fallot (TOF) [67]. Insufficient dietary folate has resulted in global DNA hypomethylation [68]. DNA hypomethylation due to multiple causes is shown to lengthen telomeres with negative regulation of TL by DNMT (DNMT1, 3A, and 3B enzymes) [69]. Hypomethylation in subtelomeric regions is expected to elicit ALT, ultimately leading to dysfunctional telomeres. Epidemiological studies indicate that folate deficiency may have a procarcinogenic effect, causing the collapse of replication forks and the accumulation of base excision repair intermediates, ultimately resulting in DNA strand breaks leading to genomic instability [69]. Similarly, excess folate is speculated to elicit TL shortening due to increased nuclear divisions [69]. Moreover, maternal hyperhomocysteinemia results in neural crest‐related developmental defects and congenital outflow tract defects (i.e., a type of CHD in offspring) [16]. Elevated homocysteine levels are one of the markers of increased oxidative stress. Embryogenesis is highly sensitive to oxidative stress, especially during the early postimplantation stage, which is marked by cardiac development. ROS mediate apoptotic cell removal during embryogenesis, whereas excess oxidative stress results in embryotoxicity. Redox homeostasis controls key cell proliferation, differentiation, necrosis, or apoptosis processes during fetal growth [70]. Any disruptions in the delicate balance of oxidants and antioxidants during embryonic development due to environmental factors will result in oxidative stress, which may explain the abnormal organogenesis. A recent study using an animal model showed that at nonphysiological oxygen levels (~20% O_2_), mouse embryos exhibited reduced telomere elongation during early cleavage stages, linking elevated oxygen exposure to altered mitochondrial regulation of telomere dynamics [71]. Together, these observations indicate that oxidative stress–mediated telomere dysregulation may represent a key mechanistic link between metabolic imbalance and embryonic developmental abnormalities.

A study by Halder et al. [72] investigated the association between folate metabolism–related gene polymorphisms and maternal nondisjunction (NDJ) errors leading to Down syndrome (DS) births in a Bengali population from eastern India. Through a large case‐control design, the researchers found that three specific polymorphisms—*MTR* A2756G, *MTRR* A66G, and *MTHFR* A1298C—are significantly associated with an increased risk of maternal meiosis II (MII) NDJ errors. Notably, DS mothers carrying these polymorphisms had a lower mean age of conception (27.3 ± 0.2 years) than those without (30.2 ± 0.4 years, *p* = 0.01), indicating age‐independent genetic susceptibility. The *MTR* A2756G variant conferred an approximately 2.9‐fold increased risk, *MTRR* A66G variant a 2.5‐fold increase, and *MTHFR* A1298C variant a 2.2‐fold risk for DS birth. The MTHFR C677T variant did not show a significant association (*p* > 0.05), underscoring ethnic genetic variability. A significant maternal haplotype (*MTHFR* 1298C‐677C; *p* = 0.0172) was also linked to MII errors. Pairwise combinations of polymorphisms (e.g., *MTR* 2756 AG–*MTRR* 66 AG) showed enhanced risk, and cumulative analysis revealed a ~15‐fold increased DS risk when all four minor alleles co‐occurred. The study suggests folate pathway gene variants are implicated in MII NDJ errors and highlights the need for mechanistic studies to confirm these molecular interactions. Another study analyzed 404 mothers of children with DS and 42 control mothers and found that DS mothers had significantly shorter telomeres, suggesting they are biologically older, regardless of chronological age. Using qPCR‐based TL assays, the study proposed that reduced TL may predispose to NDJ errors, positioning telomere shortening as a novel, age‐independent biomarker for chromosomal missegregation risk in oocytes [73].

Genetic variants in the maternal RFC1 gene, responsible for folate transport across cells, have been associated with an increased risk of CHD in offspring, a risk shown to be mitigated by folate supplementation. These findings support the need for initiating folate supplementation preconceptionally rather than during pregnancy [74]. In CHD‐affected pregnancies, altered concentrations of OCM metabolites were observed, excluding methionine, in maternal plasma (*p* < 0.001) [63]. Discrepancies were also seen between maternal and umbilical cord blood, specifically in homocysteine, cobalamin, and folate concentrations [75]. Similar patterns were reported by Hobbs et al. [76], with significantly higher maternal levels of homocysteine and SAH (*p* < 0.001) and lower levels of methionine (*p* = 0.019) and SAM (*p* = 0.014) in CHD cases versus controls. The study reported elevated median concentrations of SAM (*p* = 0.011) and both serum and RBC folate (*p* = 0.021 and *p* = 0.030, respectively) in CHD‐affected pregnancies [77]. Their data suggested that the most severe CHD phenotypes showed the highest levels of these biomarkers. Furthermore, significantly elevated SAH (*p* = 0.037) and a lower SAM:SAH ratio (*p* = 0.034) in DS cases with CHD pointed to systemic hypomethylation.

The underlying mechanisms by which folate deficiency leads to telomere shortening remain largely unclear. Figure 4 illustrates that OCM integrates the folate and methionine cycles to regulate DNA methylation, nucleotide synthesis, and redox balance, thereby maintaining genomic and telomere stability. Disruptions in this pathway elevate oxidative stress and impair methylation, leading to genomic instability, telomere dysfunction, and aberrant cellular proliferation. Based on previous studies, we can state that maternal folate deficiency, especially in the presence of folate metabolism gene polymorphisms, leads to elevated homocysteine levels, resulting in oxidative stress and SAH accumulation. If this disrupts the SAM:SAH ratio, reducing DNA methylation capacity, particularly during the early embryonic window of cardiogenesis, and consequent DNA hypomethylation, especially in subtelomeric regions, may impair telomere integrity, promoting telomere dysfunction or shortening, which is linked to chromosomal instability. These disruptions may collectively impact neural crest migration and cardiac outflow tract development, increasing the risk of CHD. Evidence supports that preconceptional folate supplementation can mitigate these risks by restoring OCM, maintaining methylation homeostasis and preventing telomere‐related developmental errors.

**Figure 4:**
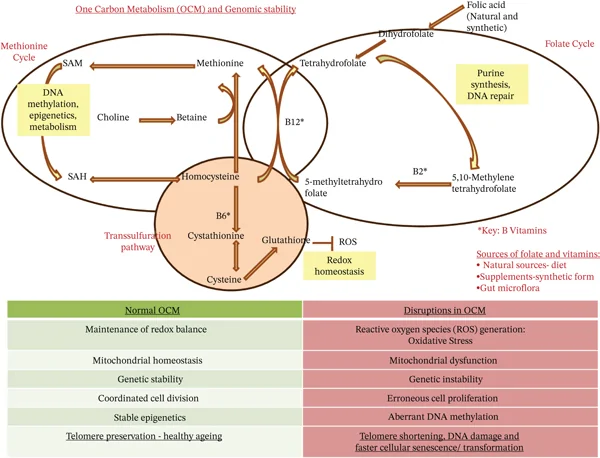
1C metabolism and effect on telomeres.

### 3.2. Future Direction

The evidence presented in this review underscores a compelling and underexplored intersection between telomere biology and the pathogenesis of CHD. Although traditionally regarded as mere markers of cellular aging, telomeres and their regulatory machinery must be further investigated as critical players in early embryonic development. Recognizing TL and epigenetic integrity as functional biomarkers of oocyte quality and embryonic viability provides a transformative lens to view CHD risk in future pregnancies. It also emphasizes the need for personalized periconceptional strategies, including folate optimization and redox balance, to preserve telomere homeostasis and ensure proper embryonic cardiac development. As the field advances, integrating telomere biology into maternal–fetal medicine holds the potential to redefine risk assessment and early intervention strategies for CHD. At the crossroads of genomics, epigenetics, and embryology, telomere biology emerges as a silent architect of cardiac fate, where even subtle maternal environmental imbalances may echo as CHD in the next generation.

**Nomenclature**
ACD: adrenocortical dysplasia protein homolog
ALT: alternative lengthening of telomeres
AMH: anti‐Müllerian hormone
ART: assisted reproductive technology
B12: vitamin B12 (cobalamin)
B9: folate (vitamin B9)
BM Failure: bone marrow failure
*BRG1*
: brahma‐related Gene 1
CDC13: cell division Cycle 13
CHD: congenital heart defect/congenital heart disease
COD: cardiac outflow defect
CST: CTC1–STN1–TEN1 complex
CTC1: conserved telomere maintenance Component 1
DC: dyskeratosis congenita
DDR: DNA damage response
DKC: dyskeratosis congenita
*DKC1*
: Dyskerin 1
DNA: deoxyribonucleic acid
DNMT1: DNA Methyltransferase 1
DS: Down syndrome
DUX4: double Homeobox 4
F0: founder generation
F2: second filial generation
FSHD: facioscapulohumeral muscular dystrophy
G0: grandparental generation
G1: first generation offspring
IVF: in vitro fertilization
kb: kilobase
LTL: leukocyte telomere length
MII: meiosis II
*MTHFR*
: methylenetetrahydrofolate reductase
*MTR*
: methionine synthase
*MTRR*
: methionine synthase reductase
NDJ: nondisjunction
OCM: one‐carbon metabolism
OR: odds ratio
PIP1: POT1‐interacting Protein 1
PMC: PubMed Central
*POT1*
: protection of Telomeres 1
PTOP: POT1 and TIN2 organizing protein
RAP1: repressor/activator Protein 1
RBC: red blood cell
RFC1: reduced folate Carrier 1
RNA: ribonucleic acid
ROS: reactive oxygen species
*RTEL1*
: regulator of telomere elongation Helicase 1
SAH: *S*‐adenosylhomocysteine
SAM: *S*‐adenosylmethionine
SASP: senescence‐associated secretory phenotype
STN1: suppressor of CDC13 homolog
STL: sperm telomere length
TEN1: telomere length regulation protein TEN1 homolog
TERC: telomerase RNA component
*TERT*
: telomerase reverse transcriptase
*TINF2*
: TERF1‐interacting nuclear Factor 2
TIN2: TRF1‐interacting nuclear Protein 2
TL: telomere length
TPE: telomere position effect
TPP1: TINT1/PTOP/PIP1 Protein 1
TRF: terminal restriction fragment
*TRF1*
: telomeric repeat binding Factor 1
*TRF2*
: telomeric repeat binding Factor 2
TTAGGG: human telomeric repeat sequence
UK: United Kingdom
VSD: ventricular septal defect
WBC: white blood cell
Wnt: wingless/integrated signaling pathway
ZGA: zygotic genome activation

## Funding

No funding was received for this manuscript.

## Ethics Statement

This article is a review of previously published research and builds a hypothesis based on the same. No new human or animal data were generated, and hence ethics approval and informed consent were not applicable.

## Conflicts of Interest

The authors declare no conflicts of interest.

## Data Availability

Data sharing not applicable to this article as no datasets were generated or analyzed during the current study.
